# *scikit-fingerprints*: A Python Library for Scikit-Learn-Compatible Molecular Fingerprints and Chemoinformatics

**DOI:** 10.1021/acs.jcim.6c02602

**Published:** 2026-10-14

**Authors:** Jakub Adamczyk, Adam Staniszewski

**Affiliations:** Faculty of Computer Science, 49811 AGH University of Krakow, Cracow 30-059, Poland

## Abstract

We present *scikit-fingerprints*, a comprehensive, fully scikit-learn-compatible library for molecular machine learning in Python, based on RDKit. Molecular fingerprints and related functionalities are workhorses of chemoinformatics, yet the widely used open-source frameworks are not compatible with the wider Python machine-learning ecosystem based on scikit-learn conventions. *scikit-fingerprints* closes this gap, bringing molecular fingerprints, molecular filters, similarity and distance measures, applicability domain estimation, data-splitting strategies, and more under a single familiar interface. Scikit-learn compatibility means that an entire chemoinformatics workflow, from a raw SMILES string to a deployable model, can be assembled from composable building blocks and can reuse the mature tooling of the surrounding ecosystem. The underlying RDKit code makes the library familiar and extensible for custom chemoinformatics use cases. We put a strong focus on unified interfaces, ease of use, computational efficiency, customization, and extensibility. *scikit-fingerprints* makes molecular machine learning faster to prototype, easier to reproduce, and simpler to deploy.

## Graphical abstract

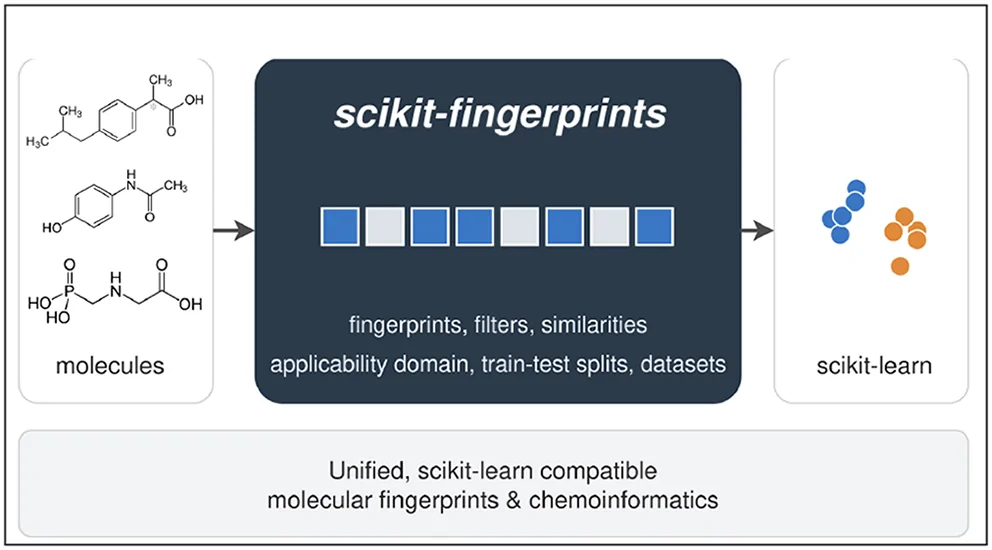

## Introduction and Motivation

Molecules are fundamental objects of chemoinformatics. They are most commonly stored as molecular graphs, which must be turned into fixed-length numerical vectors before the majority of downstream algorithms and machine learning (ML) models in particular can process them. This vectorization is typically performed with molecular fingerprints, feature-extraction algorithms that encode structural, topological, or physicochemical information about a molecule as a vector.^1^ Fingerprints underpin a broad range of chemoinformatics tasks, including molecular property and activity prediction,^2,3^ virtual screening and similarity searching,^4,5^ chemical space visualization,^6,7^ clustering and diversity picking,^8,9^ and data splitting for reliable model evaluation.^10,11^ Fingerprint-based models remain highly competitive baselines, often matching or exceeding neural networks for molecular property prediction.^2,12–15^

Building a QSAR/QSPR model, however, is rarely a single step. A realistic workflow reads and standardizes molecular structures, filters out undesirable compounds, computes one or several molecular fingerprints, splits the data to evaluate out-of-distribution generalization (e.g., via a scaffold split), tunes hyperparameters, evaluates with domain-appropriate metrics, and finally assesses whether new predictions fall within the model’s applicability domain.^16,17^ Each of these steps has a well-established methodology, yet in practice they are stitched together with ad hoc scripts, which is repetitive, error-prone, and hard to reproduce.^18^ As chemoinformatics workflows evolve from experimental scripts into production-grade deployments, there is a pressing need for proper software engineering practices and interoperability among the tools used.

In the Python ecosystem, scikit-learn^19^ has become the de facto standard for tabular machine learning, valued for its consistent and widely adopted API.^20^ A large family of compatible libraries follows the same conventions, including imbalanced-learn^21^ for resampling, UMAP^22,23^ for dimensionality reduction, and HDBSCAN^24,25^ for clustering. This shared interface is precisely what makes complex pipelines composable and reusable. Chemoinformatics, unfortunately, has largely stood apart from this. The most widely used open-source toolkits, e.g., RDKit,^26^ the Chemistry Development Kit (CDK),^27^ and Open Babel^28^, are written in C++ or Java, expose object-oriented APIs tied to their own molecule types, and provide little to no support for the scikit-learn interface and for parallel computation. Their Python wrappers can be cumbersome to combine with ML tooling, resulting in brittle pipelines that require significant custom code and duplication across QSAR/QSPR projects.

The first release of *scikit-fingerprints* addressed one part of this gap by providing efficient, scikit-learn-compatible molecular fingerprints.^29^ Since then, the library has grown well beyond fingerprinting into a general chemoinformatics toolkit, covering many functionalities applied either prior to computing molecular fingerprints or based on those representations. In this work, we present *scikit-fingerprints* 2.0, whose scope now covers the whole molecular-ML workflow inside the scikit-learn ecosystem. In addition to molecular fingerprints, the library provides molecular filters, similarity and distance measures, applicability domain estimators, scaffold-aware train-test splitters, efficient fingerprint-aware hyperparameter search, and other functionalities useful in building and evaluating QSAR/QSPR pipelines. It builds upon RDKit, with a focus on efficiency and parallelism but also conforms to the scikit-learn API. This allows users to express experimental workflows as serializable pipelines, interoperable with the rest of the ecosystem and related libraries, and easily deploy them into practical chemoinformatics applications.

*scikit-fingerprints* is fully open-source, distributed under the permissive MIT license, and is available on PyPI. The code is hosted on GitHub at https://github.com/MLCIL/scikit-fingerprints. We provide extensive documentation and tutorials on GitHub and at https://scikit-fingerprints.readthedocs.io/latest.

## Functionalities

All functionality lives in the skfp package, grouped into submodules that mirror the stages of a chemoinformatics workflow. See Figure 1 for an overview of the functionality.

**Figure 1:**
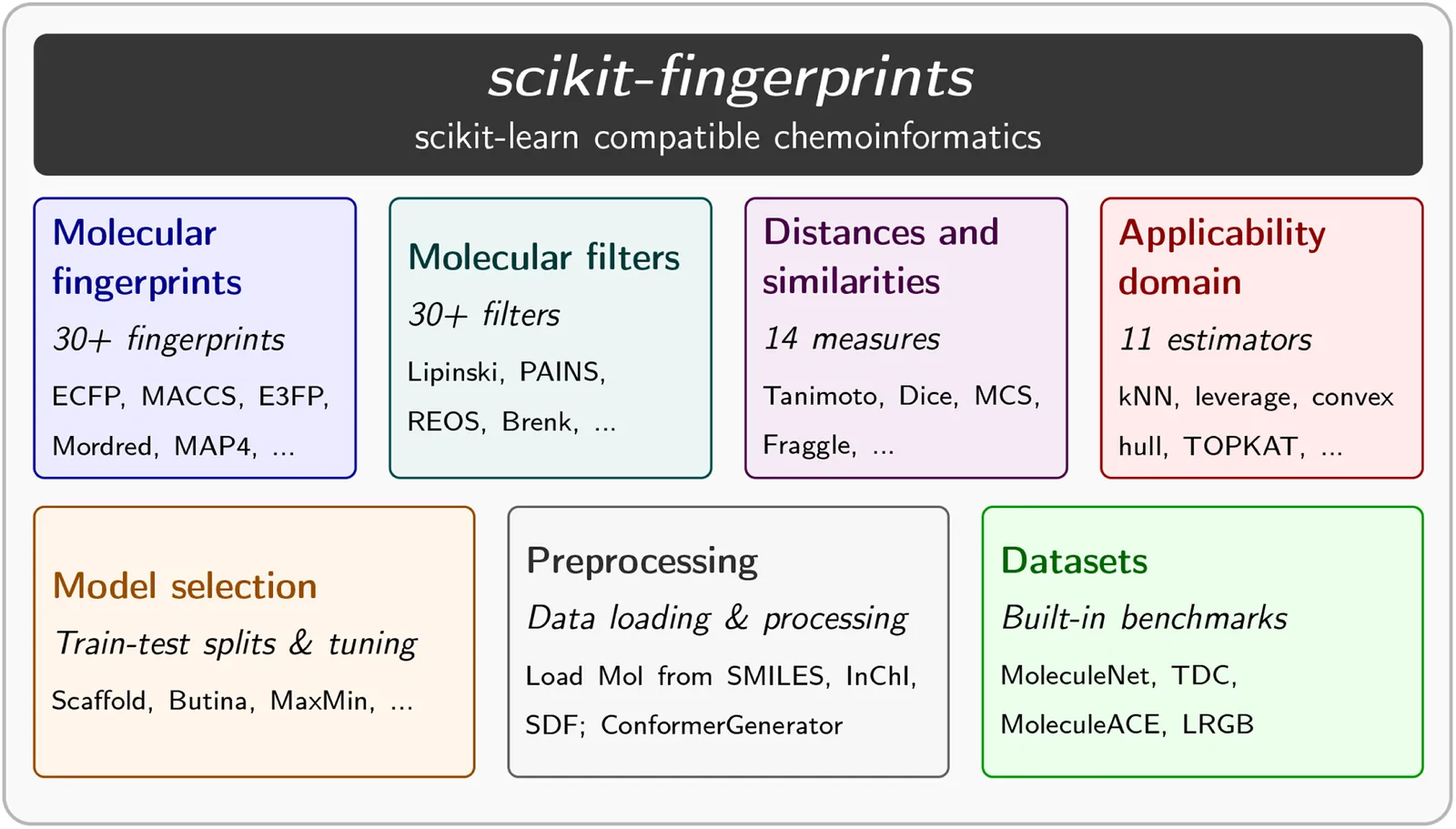
Overview of *scikit-fingerprints* functionalities.

Fingerprints and preprocessors are scikit-learn transformers, exposing the transform method. As they do not learn anything from the data, they are inherently stateless; therefore, the .fit­() method does not need to be called. All computations across different classes are parallelized with joblib, with controllable minibatching and optional progress bars. Train-test splitters, similarity and distance measures, and quality metrics are plain functions, matching the scikit-learn signatures. Compatibility with the scikit-learn API is enforced by inheritance from scikit-learn base classes and verified by a suite of unit and integration tests, run on CI/CD with a matrix of Python versions and operating systems.

We note that *scikit-fingerprints* is not merely an RDKit wrapper, but also implements a wide range of novel methods and functionalities, such as molecular filters based on physicochemical properties and applicability domain checkers. The following subsections describe each area, noting where components go beyond what RDKit provides directly. We also summarize the number of functionalities in different chemoinformatics libraries in Section Comparison with Other Software.

### Molecular Fingerprints

Molecular fingerprints are feature extraction algorithms that turn a molecule into a fixed-length numerical vector that a machine-learning model can consume. They encode structural, topological, or physicochemical information, embedding molecular graphs in a vector space, where tabular ML models can be applied.^1^ The resulting vector representation itself is also commonly termed a fingerprint.

There is a large variety of fingerprint methods since the choice of representations strongly influences downstream performance. This is particularly because the notion of molecular similarity in the resulting vector space depends on algorithmic choices. The optimal choice for a given task cannot be known in advance, a problem further complicated by the fact that fingerprints themselves can be configured via various hyperparameters. For example, a popular Extended Connectivity FingerPrint (ECFP)^30^ can optionally include chirality information or count occurrences of subgraphs rather than just detect their presence (binary vs count variants). As such, a practitioner typically wants to try many fingerprints, tune their hyperparameters, and also potentially build more complex pipelines, e.g., with feature selection or concatenation of multiple fingerprints. *scikit-fingerprints* is built around exactly this need, offering over 30 fingerprints behind a single uniform interface.

Every fingerprint is a stateless scikit-learn transformer. All fingerprint classes inherit from BaseFingerprintTransformer and accept a list of RDKit Mol objects or SMILES strings (parsed as molecules internally), returning either a dense NumPy array^31^ or a sparse SciPy CSR matrix.^32^ Behavioral options are set through constructor parameters, e.g., the output length for hashed fingerprints, or a binary vs count variant. This uniformity is the key design decision: fingerprints can be swapped or combined easily within scikit-learn pipelines. Since the transformers are stateless, not learning any parameters or fitting training data, they carry an empty .fit method and can be dropped into pipelines, cross-validation, and grid search without special handling. See Chart [Fig chart1] for a code example.

**1 chart1:**
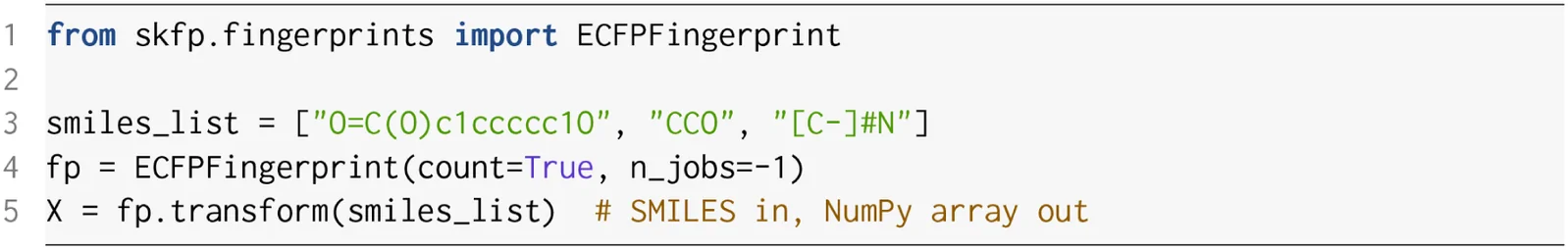
Molecular Fingerprints Example

The implemented fingerprints span all of the major families used in chemoinformatics. Substructural fingerprints, such as MACCS^33^ and Klekota-Roth,^34^ indicate the presence of predefined substructures, typically defined with SMARTS patterns, and yield directly interpretable, human-readable features. Hashed fingerprints enumerate local environments in a molecule and hash them into a vector of a chosen length. These include, for example, ECFP^30^ (also known as the Morgan fingerprint), Atom Pair,^35^ and Topological Torsion.^36^ Descriptor-based fingerprints collect scalar physicochemical or graph-theoretic quantities into a vector, ranging from electrotopological EState indices^37^ to the comprehensive Mordred descriptor set.^38^ A substantial subset of the fingerprints is conformer-based, capturing spatial geometry rather than the molecular graph alone. These include, for example, GETAWAY,^39^ E3FP,^40^ and MAP4.^41^ This coverage includes all fingerprints commonly available in established toolkits, alongside further additions, giving users a single place to obtain and compare them.

Computing fingerprints over large molecular datasets is often the most expensive step of a workflow, so efficiency is treated as a first-class concern. Because molecules are processed independently, fingerprint computation is embarrassingly parallel. Every transformer exposes an n_jobs parameter and parallelizes across CPU cores using Joblib,^42^ which also supports distributed computation on HPC clusters via a Dask backend.^43^ The underlying structural matching relies on RDKit’s optimized C++ routines.^26^ Since fingerprints are typically long and extremely sparse,^29^ particularly substructural and hashed ones, sparse output sharply reduces memory use, which is especially valuable during hyperparameter search when several copies of the feature matrix may coexist.

Following the scikit-learn convention, we assume that data fits in memory, which for sparse fingerprints allows for the processing of at least hundreds of thousands of molecules comfortably on commodity hardware. We note that memory is used primarily by RDKit Mol objects rather than by fingerprint arrays. For larger-than-memory data, we recommend using swap space on SSD drives or processing molecules manually in batches.

Interpretability is supported wherever it is meaningful. Fingerprints with human-readable features implement the standard .get_feature_names_out method; therefore, their outputs connect directly to scikit-learn feature-importance measures. As this is a standard API, it also directly integrates with external libraries and explanation tools such as SHAP,^44^ allowing model behavior to be traced back to concrete substructures or descriptors.

Some fingerprints, like Mordred,^38^ may produce missing values (NaN) for certain molecules, such as salts with disconnected components. Following the scikit-learn convention, we do not handle these internally, instead returning them as-is. This gives the user maximum flexibility, allowing the use of any imputation strategy or the removal of such features altogether.

### Molecular Filters

Molecular filters remove compounds that are undesirable for a given task, such as by retaining only drug-like compounds or removing known reactive groups. This is a standard step in many chemoinformatics workflows, particularly virtual screening and compound library design. Filters can be roughly divided into two groups: those based on allowed ranges of physicochemical properties, and those detecting disallowed substructures. The former encode primarily drug-likeness or lead-likeness rules based on simple molecular properties, including Lipinski’s Rule of Five,^45^ Ghose,^46^ Veber,^47^ and REOS,^48^ among others. We note that no such filters are implemented directly in RDKit, whereas *scikit-fingerprints* implements over a dozen. Substructural filters flag problematic chemical motifs, most notably PAINS^49^ for pan-assay interference compounds, with others including, e.g., the Brenk^50^ and Glaxo^51^ rule sets, defined with SMARTS patterns.

*scikit-fingerprints* provides over 30 filters as transformers that accept molecules and return the subset of molecules passing the rules. See Chart [Fig chart2] for a code example. As filtering is often an interactive process, detailed results of passing or failing particular filter conditions can also be returned. Since filters are often designed for specific applications, such as for pesticides^52,53^ or RNA-targeting small compounds,^54^ implementing custom ones is particularly easy. This requires only inheriting from the base filter class and overriding the _apply_mol_filter­() method.

**2 chart2:**
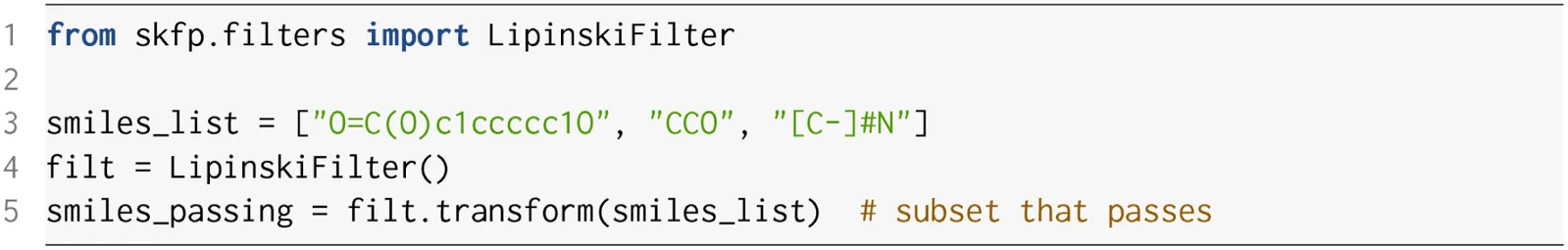
Molecular Filters Example

Filtering changes the number of samples, which the standard scikit-learn transform interface does not account for, as it returns only the features. To keep labels aligned with the retained molecules, every filter additionally provides the .transform_x_y­() method, as inspired by the feature-engine library.^55^ It accepts molecules and their labels and returns both filtered consistently. This avoids manual index bookkeeping, which is a common source of subtle errors in supervised workflows and is particularly useful when several filters are chained in sequence.

### Distances and Similarities

Similarity and distance measures are central to virtual screening, clustering, and diversity analyses. RDKit provides such measures but only for its own fingerprint objects, which prevents their direct use with the distance-based algorithms of the scikit-learn ecosystem. *scikit-fingerprints* implements them as functions, primarily based on NumPy and SciPy arrays, making them fully interoperable with scikit-learn. These include classic similarities like Tanimoto, Dice, Sokal-Sneath, or Kulczynski, as well as measures based on the direct comparison of molecular graphs, such as maximum common subgraph (MCS)^56^ and Fraggle^57^ similarities, with over a dozen measures in total. Each one is available in similarity and distance forms and, where applicable, in binary and count variants.

Because the distance functions have standard signatures, they can be passed as custom metrics to, for example, scikit-learn k-nearest neighbors, UMAP, HDBSCAN, and other methods based on nearest neighbors. This aligns the behavior of these algorithms with fingerprint representations throughout the ecosystem.

Another application is the computation of pairwise similarity matrices, which can be used as kernel matrices^58^ for SVMs and other kernel methods, as well as for diversity analysis, clustering, and other downstream tasks. We implemented a highly optimized version of such bulk computation of *N* × *N* matrices with sparse matrix operations, utilizing the fact that molecular fingerprints are highly sparse, with often just a few percent of nonzero entries.^29^ This results in significant speedups over naive loops, due to vectorization and better CPU utilization, allowing scaling to larger datasets. See Chart [Fig chart3] for a code example.

**3 chart3:**
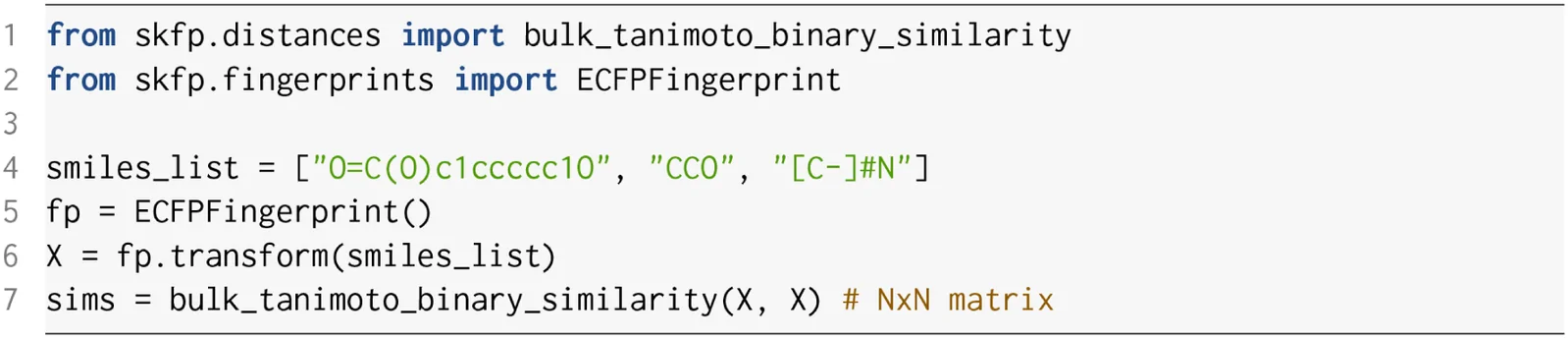
Bulk Similarity Computation Example

### Model Selection

Reliable evaluation requires splits that reflect the distribution shift a model will encounter in practice. *scikit-fingerprints* provides five scaffold-aware and diversity-aware splitting strategies as functions matching scikit-learn’s train_test_split signature: deterministic and randomized Bemis–Murcko scaffold splits,^11,59^ Butina clustering split,^8^ MaxMin diversity split,^60^ and a PubChem-based time split.^61^ Each is available in both train-test and train-validation-test forms. See Chart [Fig chart4] for a code example.

**4 chart4:**
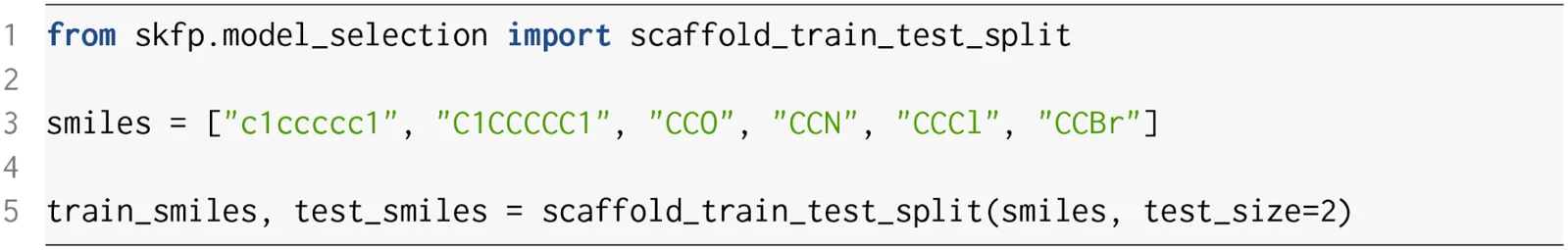
Scaffold Train-Test Split Example

Hyperparameter tuning is a common bottleneck for QSAR/QSPR pipelines because scikit-learn assumes that a .transform­() call is cheap and can be recomputed for every combination of hyperparameters. When tuning hyperparameters of molecular fingerprints and a downstream model, e.g., LightGBM,^62^ the naïve combination of Pipeline and GridSearchCV recomputes the fingerprint for every estimator configuration. *scikit-fingerprints* addresses this with a nested search loop. Two classes implement it: FingerprintEstimatorGridSearch for an exhaustive search and FingerprintEstimatorRandomizedSearch for a randomized search. In both, the fingerprint is computed once per fingerprint configuration and reused across all estimator configurations; when only estimator hyperparameters are tuned, it is computed a single time. The savings grow with the cost of the fingerprint and the size of the estimator grid, as we show in the benchmarks section.

QSAR models are frequently used for virtual screening, where the top-k ranking is of interest rather than the overall classification performance. *scikit-fingerprints* implements dedicated virtual screening measures, including enrichment factor, RIE,^63^ and BEDROC.^64^ Thanks to scikit-learn compatibility, they can, for example, be used directly as optimization targets during hyperparameter tuning.

### Preprocessing Utilities

Preprocessing transformers cover the entry and exit points of pipelines. Input–output classes convert between molecules and SMILES, InChI, SDF, and amino acid sequences, for example, MolFromSmilesTransformer and MolToInchiTransformer. Since a molecule can have many valid SMILES forms and real datasets contain questionable structures, MolStandardizer applies the widely recommended RDKit standardization steps to improve data quality at the start of a workflow. See Chart [Fig chart5] for an example.

**5 chart5:**
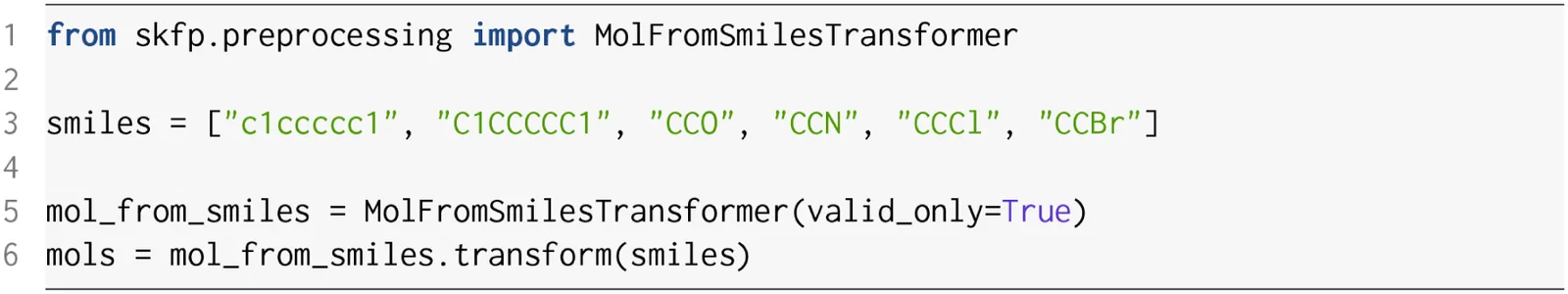
Loading Molecules from SMILES Strings

Conformer-based fingerprints require 3D structures, which can be generated with the ConformerGenerator class using the .transform­() method. It wraps the ETKDGv3 algorithm^65^ with default settings that balance speed on easy molecules against robustness on difficult molecules. Conformers for 3D fingerprints can also be provided with external tools, but our implementation is designed for easy, robust usage and is also fully parallelized.

Real-world molecular datasets frequently contain entries that RDKit cannot parse, such as malformed SMILES or hypervalent atoms that fail default sanitization. By default, such inputs raise an error, which makes problems explicit and prevents corruption of the dataset. As such cases are usually rare, preprocessing classes accept a valid_only parameter, which, when set to True, silently omits problematic molecules from the output. Because this changes the number of samples, these classes also implement the .transform_x_y method, keeping labels aligned with the retained molecules. This makes it easy to build robust ingestion pipelines over noisy data sources while retaining the strict behavior as the default.

### Applicability Domain Checks

A model’s predictions are trustworthy only for molecules resembling its training data. Applicability domain (AD) analysis quantifies this and is essential for responsible deployment, yet it has no native RDKit support. *scikit-fingerprints* implements 11 AD estimators from scratch, including distance and density approaches such as k-nearest neighbors and distance-to-centroid, geometric approaches such as bounding box, PCA bounding box, and convex hull, and statistical approaches such as leverage, Hotelling’s *T* ^2^ test, and the TOPKAT descriptor-range method.^17^

As these are dataset-dependent estimators, they implement the scikit-learn estimator interface with .fit­() and .predict­(). Fitted on training data, the estimator determines whether new molecules fall inside the domain, which is a check useful for flagging potentially unreliable predictions in production. Further, many methods provide a per-sample measure of prediction reliability, e.g., a leverage value,^66^ and they can be computed using the .score_samples­() method.

### Built-In Datasets

Fair benchmarking depends on standardized datasets and splits. *scikit-fingerprints* hosts curated datasets on the HuggingFace Hub^67^ with automatic downloading, caching, and Parquet-based compression, loaded through scikit-learn-style functions such as load_bbbp.

The current version integrates the widely used MoleculeNet benchmark,^10^ the Therapeutics Data Commons (TDC),^68^ the Long Range Graph Benchmark (LRGB) peptide datasets,^69^ and MoleculeACE for activity-cliff evaluation.^70^ Data can be returned as SMILES and labels or as a Pandas DataFrame.^71^ Predefined train-test splits are also provided, where possible, for standardized benchmarking. See Chart [Fig chart6] for a code example.

**6 chart6:**
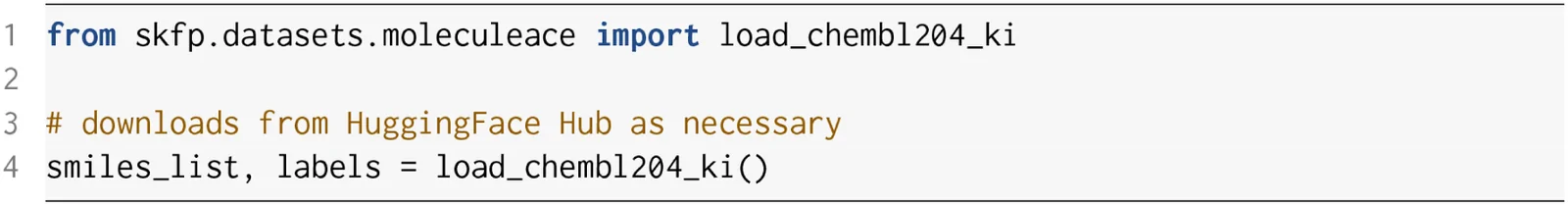
Example of Loading a Built-in MoleculeACE ChEMBL 204 Ki dataset

## Comparison with Other Software

There are several alternative open-source packages that perform similar functions to those of scikit-fingerprints. We summarize these functionalities in Table 1. Overall, scikit-fingerprints offers the widest range of capabilities, combining the largest collection of molecular fingerprints with molecular filters, applicability domain checkers, and integrated benchmark datasets, which are not jointly available elsewhere.

**Table 1:** Comparison of Capabilities of Different Chemoinformatics Software

	CDK	Open Babel	RDKit	scikit-mol	MolPipeline	scikit-fingerprints
Language	Java	C++	C++	Python	Python	Python
pip-installable	No	Yes	Yes	Yes	Yes	Yes
Last PyPI update	Never	2026	2026	2025	2026	2026
Fingerprints	13	7	25	8	6	34
Molecular filters	0	0	15	0	0	31
Applicability domain checkers	0	0	0	11	0	11
Integrated datasets	No	No	No	No	No	Yes
scikit-learn compatible	No	No	No	Yes	Yes	Yes
Parallelism	No	Yes	Partial	Yes	Yes	Yes
Easy proprietary usage	Yes (LGPL-2.1)	No (GPL-2.0)	Yes (BSD-3-Clause)	Yes (LGPL-3.0)	Yes (MIT)	Yes (MIT)

CDK^27^ and Open Babel^28^ are mature and feature-rich toolkits, but they are not compatible with scikit-learn. Further, CDK is Java-based and unavailable on PyPI, while Open Babel’s copyleft GPL-2.0 license restricts use in proprietary settings. RDKit^26^ is the most comprehensive general-purpose toolkit and serves as the computational backend of scikit-fingerprints, but its object-oriented C++ API is not scikit-learn-compatible, and it provides neither applicability domain analysis nor curated datasets. Among the Python-native, scikit-learn-compatible libraries, scikit-mol^72^ and MolPipeline^16^ share our design philosophy, but address narrower parts of the workflow, with considerably fewer fingerprints and no filters or integrated datasets. scikit-fingerprints is therefore the first package to cover the full QSAR/QSPR workflow under a single permissively licensed and fully scikit-learn-compatible interface.

## Usage Examples

Here, we present larger usage examples as the value of unified interfaces is greatest in end-to-end pipelines. In particular, full chemoinformatics workflows can be easily implemented with *scikit-fingerprints*, covering various tasks, such as QSAR/QSPR, virtual screening, visualization, and more.

In Chart [Fig chart7], we present a complete classification pipeline for the hERG dataset by Karim et al.^73^ from the Therapeutics Data Commons (TDC) benchmark.^68^ This dataset is a binary classification task, with molecules labeled as hERG (<10 μM) and non-hERG (≥10 μM) blockers. We load the data (automatically downloading it from HuggingFace Hub), convert SMILES to molecules, perform a scaffold train-test split, compute count-based ECFP fingerprints, train a Random Forest classifier, and evaluate using the AUROC metric. The entire workflow integrates directly with scikit-learn as a Pipeline object, which is finally retrained on the whole dataset and serialized to disk with Joblib. It could then be deployed to a production QSAR application to serve predictions via a web server, for example.

**7 chart7:**
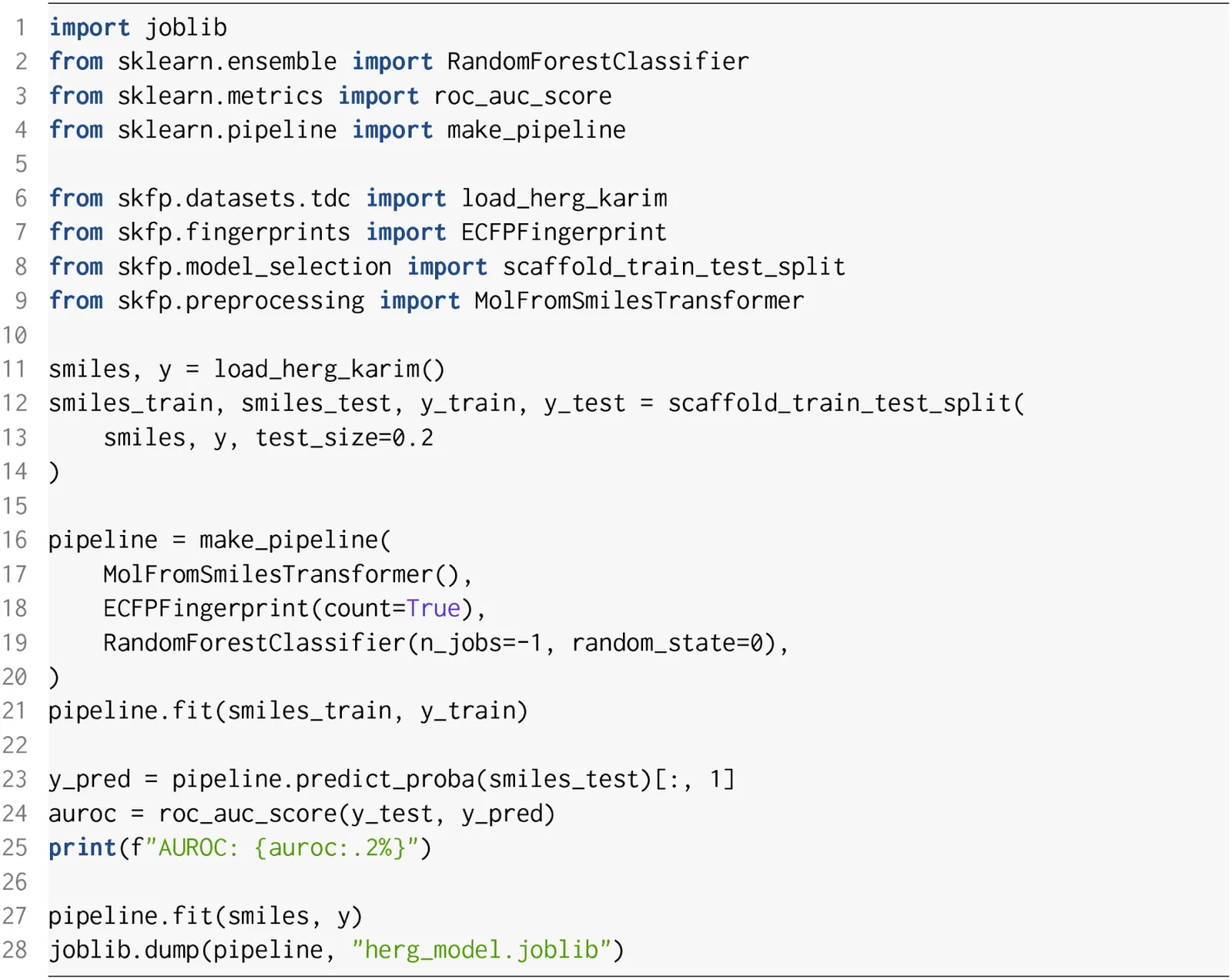
End-to-End QSAR hERG Classification Pipeline

As a more involved pipeline, in Chart [Fig chart8] we present a conformer-based QSAR pipeline for regression, which also integrates with other libraries in the scikit-learn ecosystem. We first load a generic CSV file with “SMILES” and “target” columns. Then we generate conformations with the ETKDGv3 algorithm, perform a Butina train–test split, compute and concatenate E3FP fingerprints and RDKit 2D descriptors, remove highly correlated features with feature-engine,^55^ train a LightGBM regressor,^62^ and evaluate using the MAE metric.

**8 chart8:**
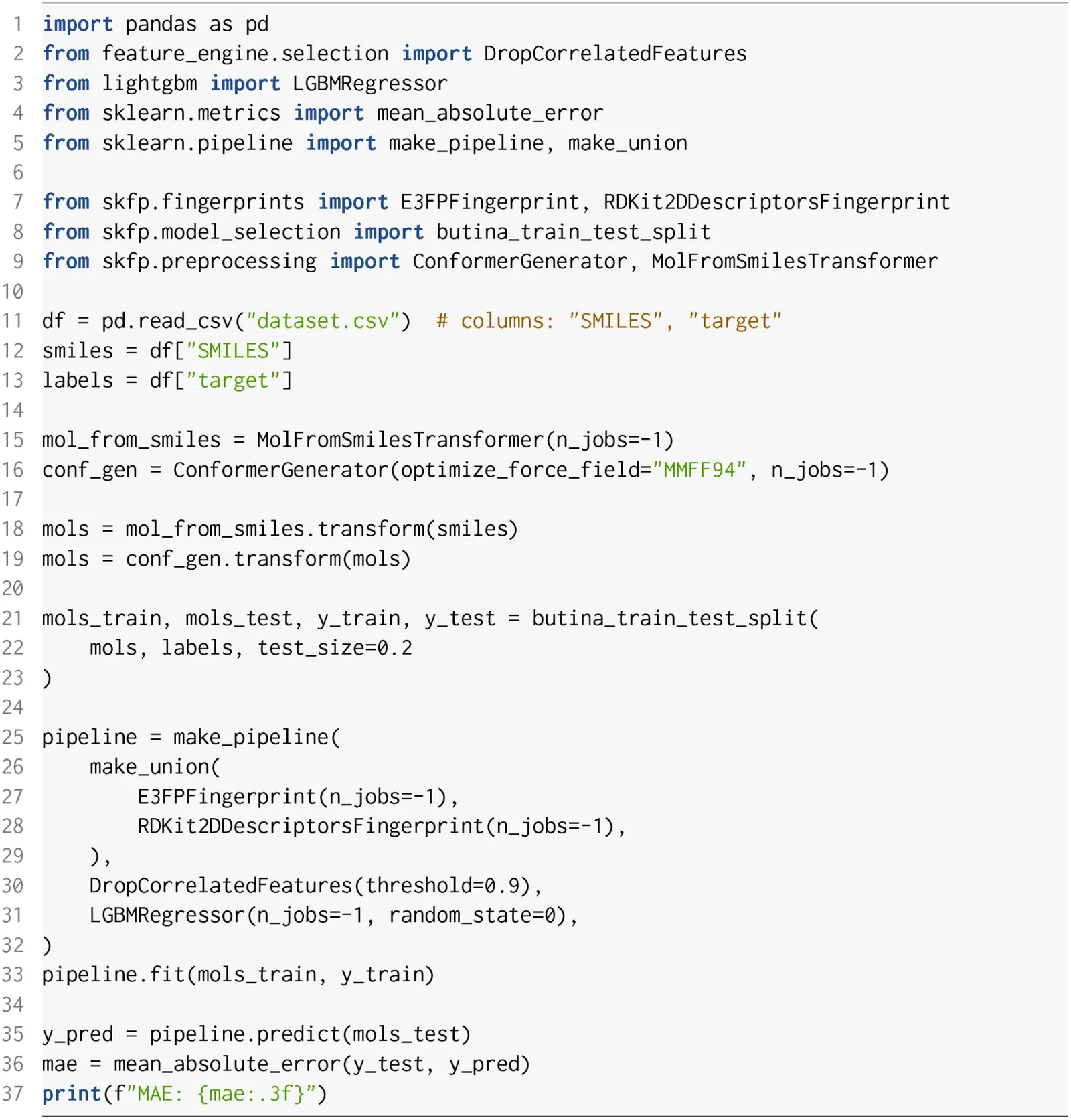
Conformer-Based QSAR Regression

Ligand-based virtual screening pipelines are commonly applied to filter large libraries. *scikit-fingerprints* offers parallelization with minibatching, molecular filters, sparse fingerprints for memory usage reduction, and other functionalities useful for this purpose. In Chart [Fig chart9], we present such a pipeline for a generic bioactivity screening, modeled as binary classification. We apply REOS and PAINS filters,^48,49^ perform a MaxMin split,^60^ calculate sparse PubChem fingerprints,^74^ train a logistic regression classifier using a fast solver for sparse matrices, and finally evaluate using the BEDROC metric,^64^ which checks early enrichment capabilities. This pipeline is applied directly to SMILES, creating RDKit Mol objects internally in minibatches. This minimizes memory usage as, at scale, these objects take up significantly more memory than SMILES strings.

**9 chart9:**
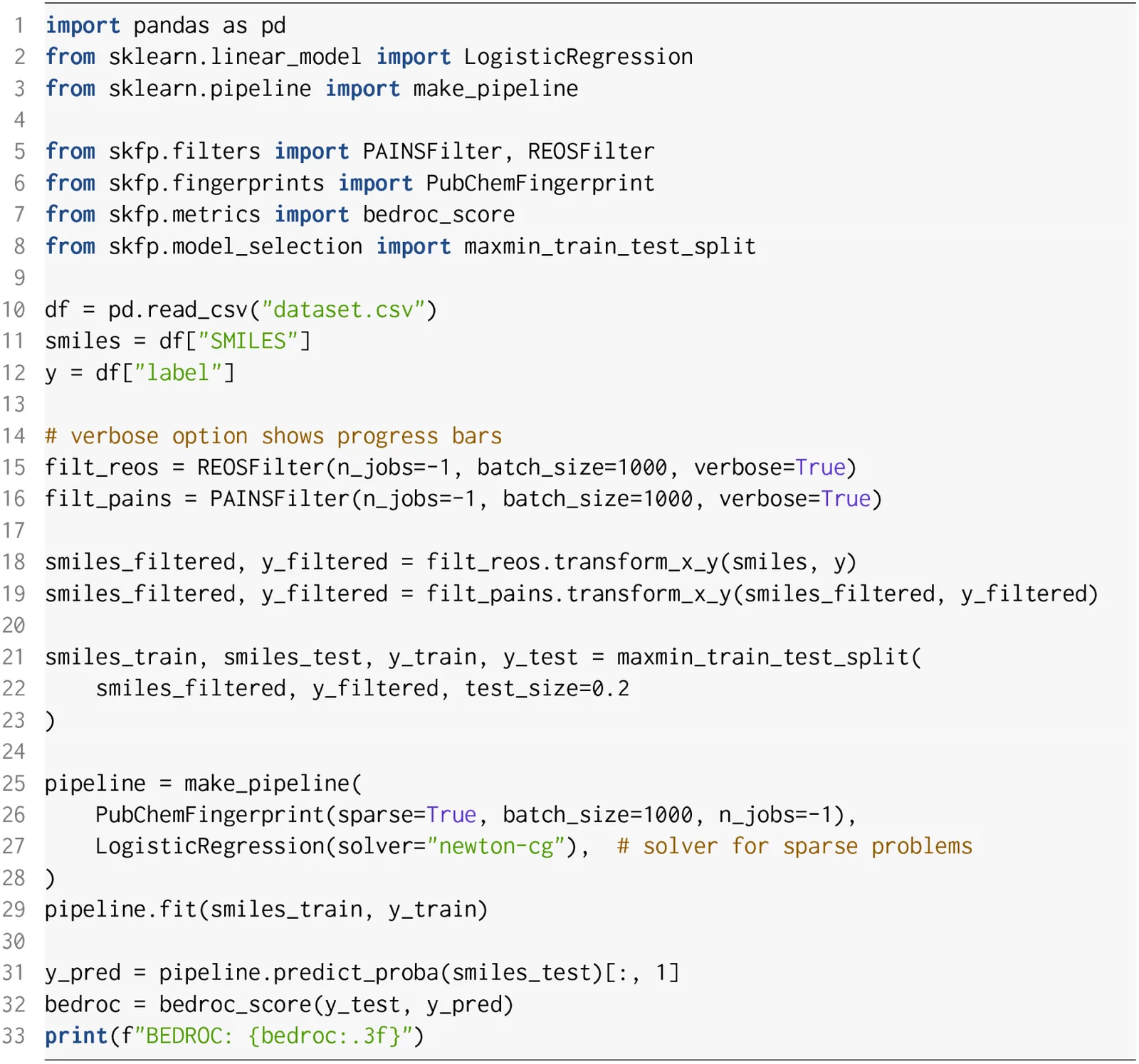
Ligand-Based Virtual Screening Pipeline

Lastly, in Chart [Fig chart10] we show a data exploration scenario, where the goal is to visualize a molecular dataset on a 2D plane using UMAP.^22,23^ *scikit-fingerprints* offers distances and similarities based on NumPy arrays with interfaces integrating with the rest of the scikit-learn ecosystem. We load the BACE dataset from the MoleculeNet benchmark,^10^ compute count-based ECFP fingerprints, and then pass the Tanimoto distance for count vectors^75^ to UMAP for dimensionality reduction.

**10 chart10:**
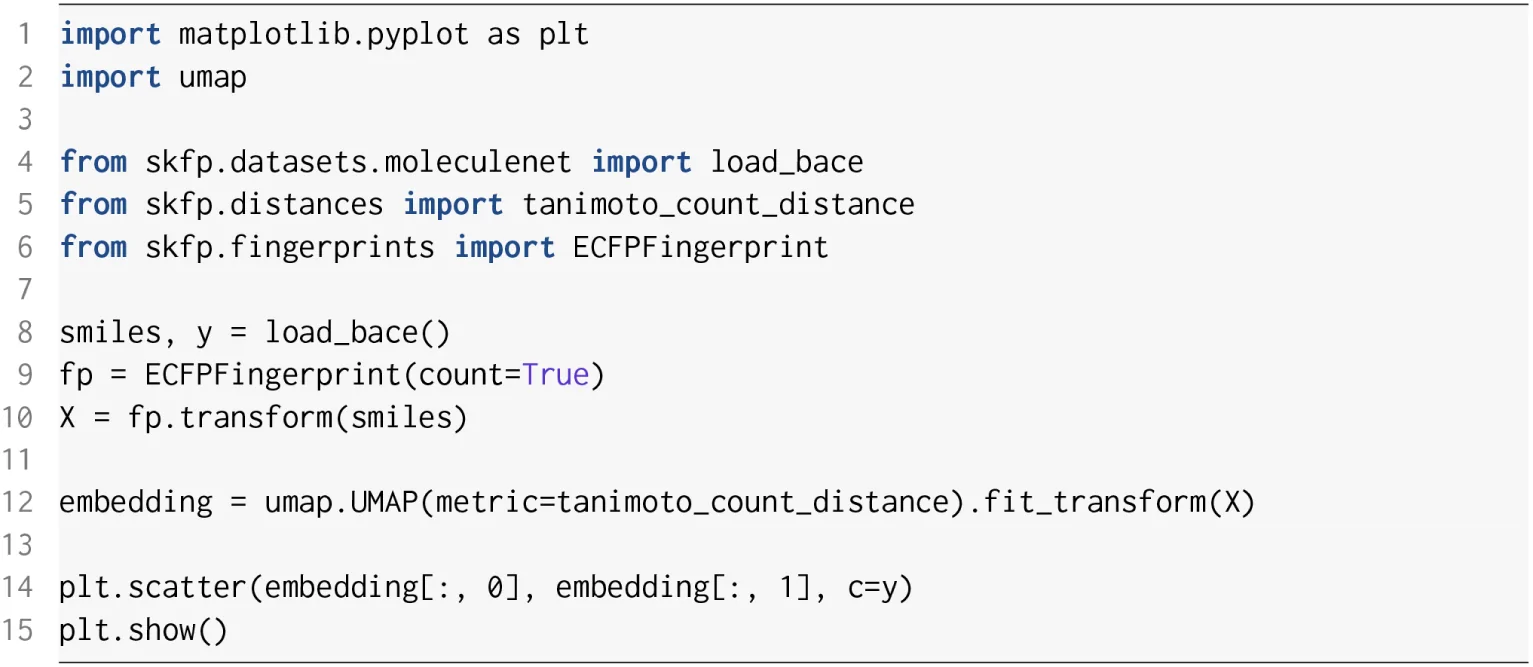
Molecular Dataset Visualization

## Benchmarks

We evaluated *scikit-fingerprints* in a few selected use cases. All experiments in this section are based on the representative HIV dataset of the MoleculeNet benchmark.^10^ It was chosen since it is large enough (over 41000 compounds) to evaluate scalability on typical QSAR/QSPR workflows. Experiments were performed on a PC with an Intel Core i9-13900H CPU (14 cores, 20 threads) and 64 GB of RAM. All reported measurements are averages of 5 runs.

### Hyperparameter Tuning

As described in the model selection section, *scikit-fingerprints* implements the FingerprintEstimatorGridSearch class. Whereas the typical combination of GridSearchCV and Pipeline in scikit-learn recomputes fingerprints for every parameter combination and cross-validation fold, our implementation optimizes this, by reusing fingerprints computed for given hyperparameters when tuning downstream classifiers. Gains are particularly strong when tuning fingerprints that are more expensive to compute and with larger grids of downstream models.

We use a combination of an RDKit fingerprint^26^ and a Random Forest (RF) classifier, with two hyperparameter grids for tuning:

- small: fingerprint {“count”: [False, True], “fp_size”: [1024, 2048] }, Random Forest {“min_samples_split”: [2, 5, 10] }
- large: fingerprint {“count”: [False, True], “fp_size”: [512, 1024, 2048] }, Random Forest {“min_samples_split”: [2, 5, 10], “max_depth”: [None, 10, 20], “n_estimators”: [100, 200, 500] }

For the small grid, this results in 4 combinations for the fingerprint and 3 for RF, so with 5-fold CV the scikit-learn implementation computes the fingerprint 60 times, and *scikit-fingerprints* only 4 times. For the large grid, there are 6 and 27 combinations, respectively, which give 810 fingerprint computations for scikit-learn and 6 for *scikit-fingerprints*.

Figure 2 shows total tuning time as a function of dataset size. Our implementation is faster in every case, but the gains are stronger for the large grid as expected. For 10000 molecules, *scikit-fingerprints* is faster by almost 9.5 min.

**Figure 2:**
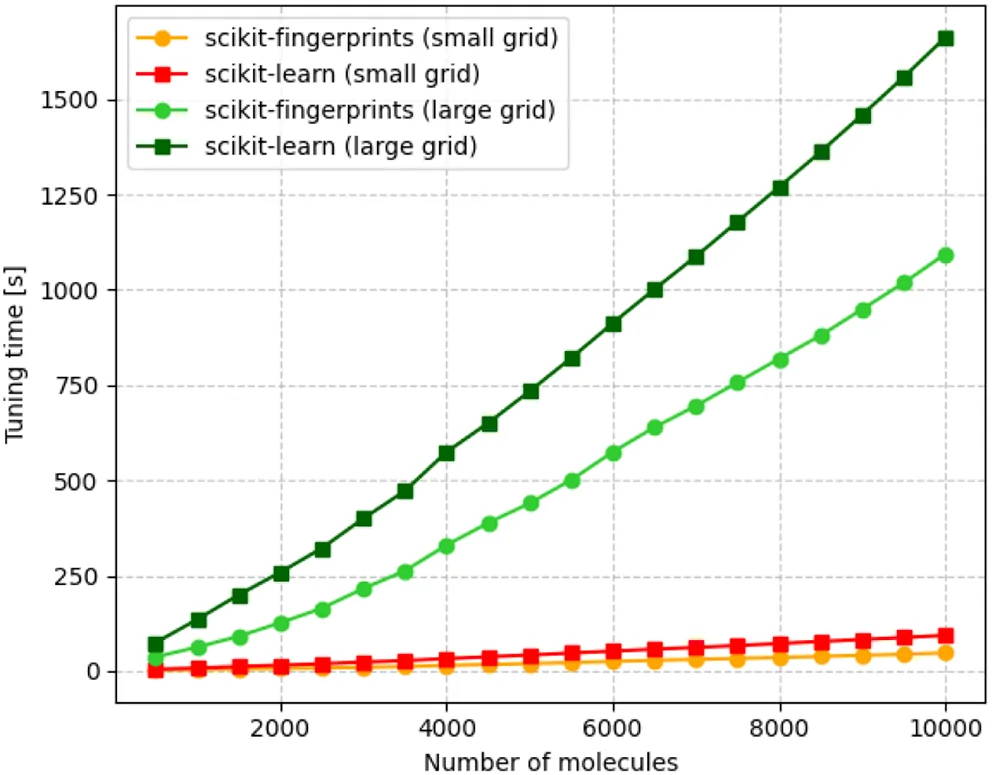
Hyperparameter tuning time.

### PubChem Fingerprint Calculation

The PubChem (CACTVS) fingerprint^74^ is an 881-bit substructure-key fingerprint, designed for similarity searching, and exposed through the PUG REST Web API. However, this implementation has considerable downsides: it works only for compounds in PubChem, requires first fetching the CID number based on SMILES, requires a large number of HTTP requests, is frequently overloaded and returns errors requiring retries, and allows only limited parallelization per client due to rate-limiting. Together, these result in very high computational time to embed whole datasets.

As key definitions are publicly available, we implemented them in the PubChemFingerprint class, using SMARTS patterns. Our implementation is also fully parallelized, and we also offer a count-based variant, as many binary keys rely on counting atoms, e.g., the number of carbons *C* ≥ 2, *C* ≥ 4, *C* ≥ 8, *C* ≥ 16. Our local implementation is considerably faster, primarily thanks to no network latency, as shown in Figure 3, allowing scaling to much larger datasets. We measure only the time to calculate fingerprints based on CID numbers, so in practice the total API time would be roughly twice as long.

**Figure 3:**
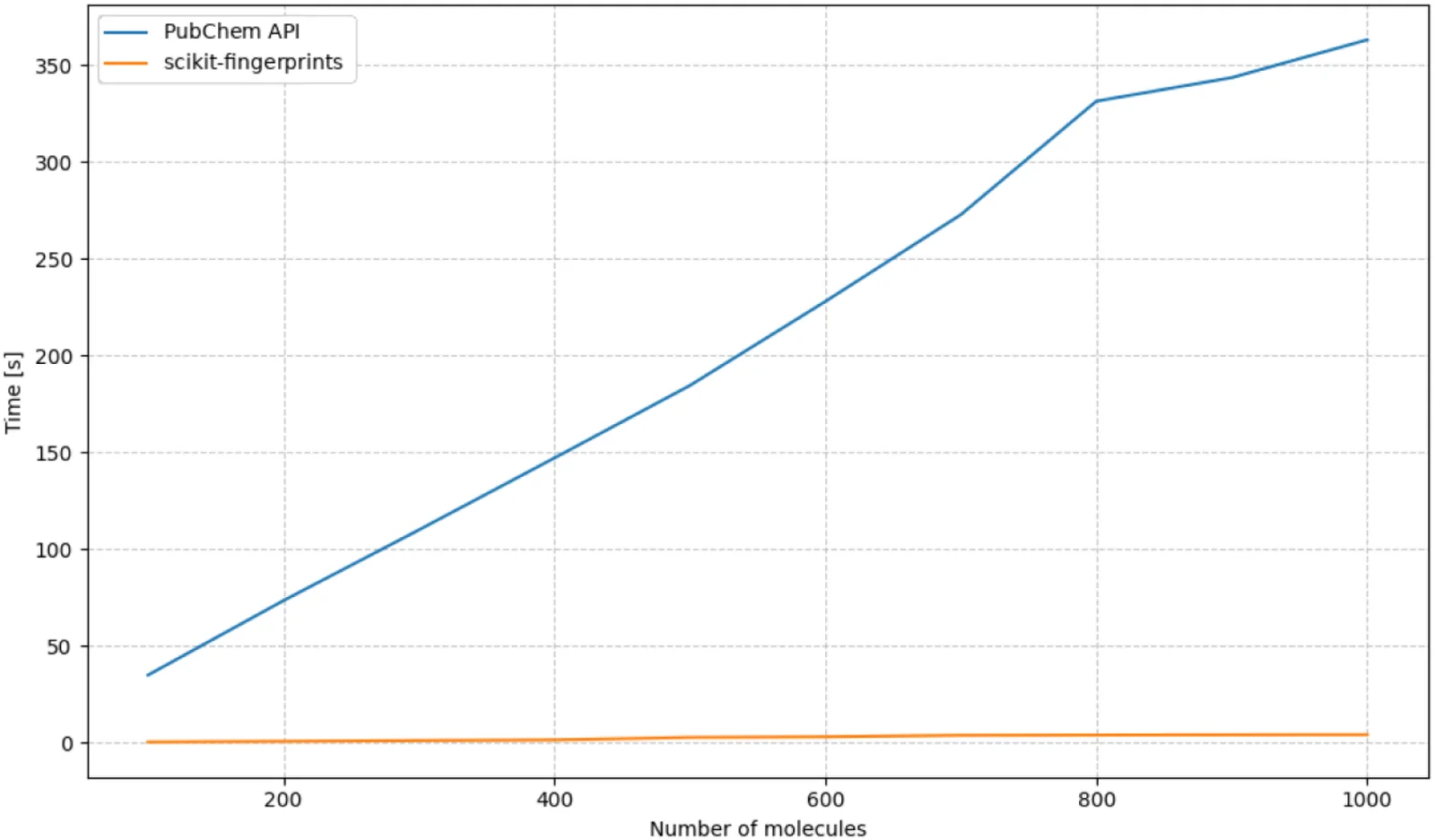
PubChem fingerprint computation.

### Bulk Tanimoto Similarity Computation

Computing bulk Tanimoto similarities between molecules, resulting in an *N* × *K* similarity matrix, arises in many places in chemoinformatics. Examples include evaluating the similarity of known active ligands to compounds in a screening catalog, or visualizing the distribution of similarities between molecules in a dataset (resulting in an *N* × *N* matrix) or between datasets. As molecular fingerprints are very sparse, and most similarity measures for binary vectors use only bit-wise AND/OR/SUM operations, such bulk computation can be implemented using sparse CSR matrix operations. Such an approach is not available in RDKit, which only supports one-to-many similarity evaluations, resulting in function-call overhead.

In Figure 4, we compare the speed of *scikit-fingerprints* and RDKit for bulk *N* × *N* similarity matrix computation. Our implementation is considerably faster for this type of task, with favorable scalability particularly for larger data sizes.

**Figure 4:**
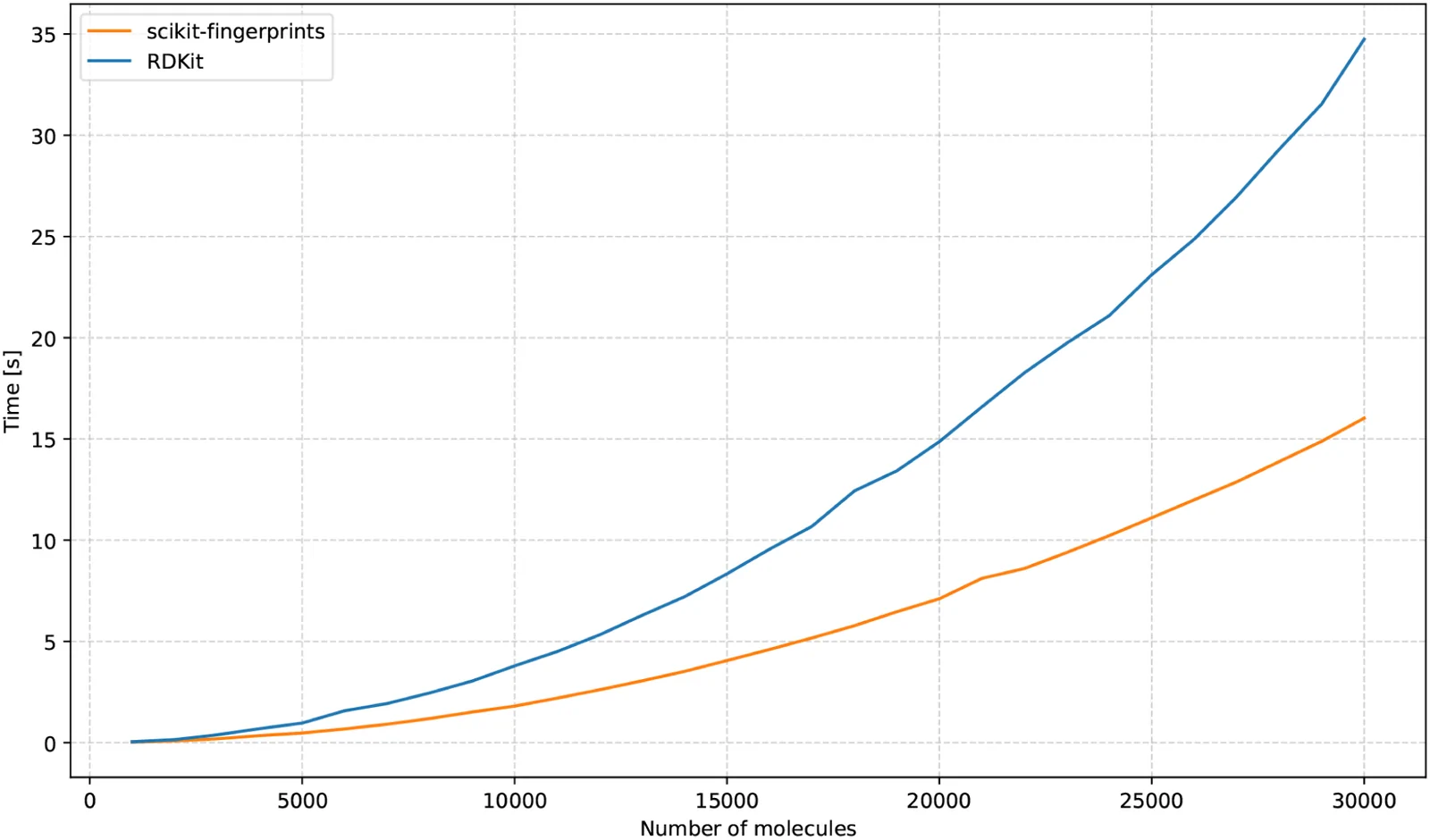
Bulk Tanimoto similarity computation.

### Large-Scale Fingerprint Computation

Molecular fingerprints are nowadays used for the exploration and virtual screening of large-scale chemical spaces. For such use cases, parallelization, minibatching, and sparse representations in *scikit-fingerprints* are directly useful. As an example, we calculate 2048-bit ECFP4 fingerprints on COCONUT,^76^ ChEMBL 37,^77^ and Mcule (In Stock subset).^78^

We measured the total computation time, molecules per second (throughput), and total memory usage when using sparse fingerprints. To contextualize the gain from using the latter, we also calculated how much memory the dense representation would use and the resulting memory savings. Results are summarized in Table 2. *scikit-fingerprints* is highly scalable to such libraries even on commodity hardware, in terms of both wall time and RAM requirements.

**Table 2:** Large-Scale Fingerprint Computation

Dataset	Number of molecules	Total time	Molecules per second	Sparse matrix memory	Dense matrix memory	Memory usage reduction ratio
COCONUT	695 k	15 s	46.3 k	35 MB	1.3 GB	39×
ChEMBL	2.9 M	62 s	46.7 k	145 MB	5.5 GB	39×
Mcule	7.1 M	1534 s	4.7 k	312 MB	13.6 GB	45×

## Conclusion

*scikit-fingerprints* brings the breadth of chemoinformatics to the scikit-learn ecosystem. Building on RDKit, it unifies molecular fingerprints, filters, distance and similarity measures, applicability domain estimation, scaffold- and diversity-aware data splitting, fingerprint-aware hyperparameter tuning, virtual screening metrics, and curated benchmark datasets behind a single, consistent interface. Because every component is a scikit-learn transformer or a compatible function, an entire workflow, from a raw SMILES string to a serialized, deployable model, can be expressed as a composable pipeline and combined freely with the surrounding ecosystem. Alongside this uniformity, our focus on parallelization, minibatching, and sparse representations keeps the library efficient, while its extensible design makes adding custom components straightforward.

*scikit-fingerprints* is under active development, with new functionalities further expanding the depth and scope of the library. We are working toward integrating embeddings from pretrained neural networks, like CLAMP^79^ and CheMeleon,^80^ as molecular fingerprints based on representation learning.^81^ Other directions include adding clustering, such as Taylor-Butina,^8^ and diversity picking like Ashton’s algorithm,^60^ as well as further train-test splitting strategies like DataSAIL.^82^ As a fully open-source project under the permissive MIT license, *scikit-fingerprints* welcomes community contributions, and we hope it will continue to lower the barrier to reproducible, production-ready molecular machine learning.

## Data and Software Availability

All data used is publicly available and hosted on HuggingFace Hub at https://huggingface.co/scikit-fingerprints/datasets. *scikit-fingerprints* is a free opensource software under MIT license, available on GitHub: https://github.com/MLCIL/scikit-fingerprints.

## References

[1] Todeschini, R. ; Consonni, V. Molecular Descriptors for Chemoinformatics; Wiley, 2009. DOI:10.1002/9783527628766

[2] Jiang D., Wu Z., Hsieh C.-Y., Chen G., Liao B., Wang Z., Shen C., Cao D., Wu J., Hou T. (2021). Could Graph
Neural Networks Learn Better Molecular Representation for Drug Discovery?
A Comparison Study of Descriptor-Based and Graph-Based Models. J. Cheminf..

[3] Zagidullin B., Wang Z., Guan Y., Pitkänen E., Tang J. (2021). Comparative Analysis of Molecular
Fingerprints in Prediction of Drug
Combination Effects. Briefings Bioinf..

[4] Riniker S., Landrum G. A. (2013). Open-Source Platform to Benchmark Fingerprints for
Ligand-Based Virtual Screening. J. Cheminf..

[5] Muegge I., Mukherjee P. (2016). An Overview of Molecular Fingerprint
Similarity Search
in Virtual Screening. Expert Opin. Drug Discovery.

[6] Riniker S., Landrum G. A. (2013). Similarity Maps
- A Visualization Strategy for Molecular
Fingerprints and Machine-Learning Methods. J.
Cheminf..

[7] Lovrić M., Duričić T., Tran H. T. N., Hussain H., Lacić E., Rasmussen M. A., Kern R. (2021). Should We Embed in
Chemistry? A Comparison of Unsupervised Transfer Learning with PCA,
UMAP, and VAE on Molecular Fingerprints. Pharmaceuticals.

[8] Butina D. (1999). Unsupervised
Data Base Clustering Based on Daylight’s Fingerprint and Tanimoto
Similarity: A Fast and Automated Way to Cluster Small and Large Data
Sets. J. Chem. Inf. Comput. Sci..

[9] Hernández-Hernández S., Ballester P. J. (2023). On the Best Way to Cluster NCI-60 Molecules. Biomolecules.

[10] Wu Z., Ramsundar B., Feinberg E. N., Gomes J., Geniesse C., Pappu A. S., Leswing K., Pande V. (2018). MoleculeNet: A Benchmark
for Molecular Machine Learning. Chem. Sci..

[11] Hu, W. ; Fey, M. ; Zitnik, M. ; Dong, Y. ; Ren, H. ; Liu, B. ; Catasta, M. ; Leskovec, J. Open Graph Benchmark: Datasets for Machine Learning on Graphs. In Proceedings of the 34th International Conference on Neural Information Processing Systems; ACM, 2020, 33, 22118-22133.

[12] Adamczyk J., Czech W. (2024). Molecular Topological Profile (MOLTOP) - Simple and Strong Baseline
for Molecular Graph Classification. Front. Artificial
Intell. Appl..

[13] Adamczyk J., Ludynia P., Czech W. (2026). Molecular
fingerprints are strong
models for peptide function prediction. Bioinformatics.

[14] Adamczyk J., Poziemski J., Siedlecki P. (2026). Evaluating machine learning models
for predicting pesticide toxicity to honey bees. Ecotoxicol. Environ. Saf..

[15] Adamczyk, J. Towards Rational Pesticide Design with Graph Machine Learning Models for Ecotoxicology. In Proceedings Of The 34th ACM International Conference On Information And Knowledge Management; ACM, 2025, pp.6777–6780. DOI: 10.1145/3746252.3761660

[16] Sieg J., Feldmann C. W., Hemmerich J., Stork C., Sandfort F., Eiden P., Mathea M. (2024). MolPipeline:
A Python Package for
Processing Molecules with RDKit in Scikit-learn. J. Chem. Inf. Model..

[17] Sahigara F., Mansouri K., Ballabio D., Mauri A., Consonni V., Todeschini R. (2012). Comparison of Different Approaches
to Define the Applicability
Domain of QSAR Models. Molecules.

[18] Hoyt C. T., Zdrazil B., Guha R., Jeliazkova N., Martinez-Mayorga K., Nittinger E. (2023). Improving
Reproducibility and Reusability
in the Journal of Cheminformatics. J. Cheminf..

[19] Pedregosa, F. . Scikit-learn: Machine Learning in Python. arXiv. 2011.

[20] Buitinck, L. ; Louppe, G. ; Blondel, M. ; Pedregosa, F. ; Mueller, A. ; Grisel, O. ; Niculae, V. ; Prettenhofer, P. ; Gramfort, A. ; Grobler, J. ; . API Design for Machine Learning Software: Experiences from the Scikit-learn Project. arXiv, 2013.

[21] Lemaitre, G. ; Nogueira, F. ; Aridas, C. K. Imbalanced-learn: A Python Toolbox to Tackle the Curse of Imbalanced Datasets in Machine Learning. arXiv. 2017.

[22] McInnes, L. ; Healy, J. ; Melville, J. UMAP: Uniform Manifold Approximation and Projection for Dimension Reduction. arXiv. 2018.

[23] Healy J., McInnes L. (2024). Uniform manifold approximation
and projection. Nat. Rev. Methods Primers.

[24] McInnes L., Healy J., Astels S. (2017). hdbscan: Hierarchical
Density Based
Clustering. J. Open Source Software.

[25] McInnes, L. ; Healy, J. Accelerated Hierarchical Density Based Clustering. Data Mining Workshops (ICDMW), 2017 IEEE International Conference On; IEEE, 2017, 33–42.

[26] Landrum, G. , RDKit: Open-Source Cheminformatics. https://www.rdkit.org, 2026.

[27] Steinbeck C., Han Y., Kuhn S., Horlacher O., Luttmann E., Willighagen E. (2003). The Chemistry
Development Kit (CDK): An Open-Source Java Library for Chemo- and
Bioinformatics. J. Chem. Inf. Comput. Sci..

[28] O’boyle N. M., Banck M., James C. A., Morley C., Vandermeersch T., Hutchison G. R. (2011). Open Babel:
An Open Chemical Toolbox. J. Cheminf..

[29] Adamczyk J., Ludynia P. (2024). Scikit-fingerprints: Easy and Efficient
Computation
of Molecular Fingerprints in Python. SoftwareX.

[30] Rogers D., Hahn M. (2010). Extended-Connectivity
Fingerprints. J. Chem.
Inf. Model..

[31] Harris C.
R., Millman K. J., van der Walt S. J., Gommers R., Virtanen P., Cournapeau D., Wieser E., Taylor J., Berg S., Smith N. J. (2020). Array Programming with NumPy. Nature.

[32] Virtanen P., Gommers R., Oliphant T. E., Haberland M., Reddy T., Cournapeau D., Burovski E., Peterson P., Weckesser W., Bright J. (2020). SciPy 1.0: Fundamental
Algorithms for Scientific Computing in Python. Nat. Methods.

[33] Durant J. L., Leland B. A., Henry D. R., Nourse J. G. (2002). Reoptimization of
MDL Keys for Use in Drug Discovery. J. Chem.
Inf. Comput. Sci..

[34] Klekota J., Roth F. P. (2008). Chemical Substructures That Enrich for Biological Activity. Bioinformatics.

[35] Carhart R. E., Smith D. H., Venkataraghavan R. (1985). Atom Pairs
as Molecular Features
in Structure-Activity Studies: Definition and Applications. J. Chem. Inf. Comput. Sci..

[36] Nilakantan R., Bauman N., Dixon J. S., Venkataraghavan R. (1987). Topological
Torsion: A New Molecular Descriptor for SAR Applications. Comparison
with Other Descriptors. J. Chem. Inf. Comput.
Sci..

[37] Hall L. H., Kier L. B. (1995). Electrotopological State Indices for Atom Types: A
Novel Combination of Electronic, Topological, and Valence State Information. J. Chem. Inf. Comput. Sci..

[38] Moriwaki H., Tian Y.-S., Kawashita N., Takagi T. (2018). Mordred: A Molecular
Descriptor Calculator. J. Cheminf..

[39] Consonni V., Todeschini R., Pavan M. (2002). Structure/Response
Correlations and
Similarity/Diversity Analysis by GETAWAY Descriptors. 1. Theory of
the Novel 3D Molecular Descriptors. J. Chem.
Inf. Comput. Sci..

[40] Axen S. D., Huang X.-P., Cáceres E. L., Gendelev L., Roth B. L., Keiser M. J. (2017). A Simple Representation of Three-Dimensional Molecular
Structure. J. Med. Chem..

[41] Capecchi A., Probst D., Reymond J.-L. (2020). One Molecular
Fingerprint to Rule
Them All: Drugs, Biomolecules, and the Metabolome. J. Cheminf..

[42] Joblib Development Team Joblib: Running Python Functions as Pipeline Jobs. https://joblib.readthedocs.io, 2024.

[43] Rocklin, M. Dask: Parallel Computation with Blocked Algorithms and Task Scheduling. In Proceedings Of The 14th Python In Science Conference; SciPy, 2015.

[44] Lundberg, S. M. ; Lee, S.-I. A Unified Approach to Interpreting Model Predictions. Advances In Neural Information Processing Systems; Curran Associates, Inc. 2017.

[45] Lipinski C. A., Lombardo F., Dominy B. W., Feeney P. J. (1997). Experimental
and
Computational Approaches to Estimate Solubility and Permeability in
Drug Discovery and Development Settings. Adv.
Drug Delivery Rev..

[46] Ghose A. K., Viswanadhan V. N., Wendoloski J. J. (1999). A Knowledge-Based
Approach in Designing
Combinatorial or Medicinal Chemistry Libraries for Drug Discovery.
1. A Qualitative and Quantitative Characterization of Known Drug Databases. J. Comb. Chem..

[47] Veber D. F., Johnson S. R., Cheng H.-Y., Smith B. R., Ward K. W., Kopple K. D. (2002). Molecular Properties That Influence the Oral Bioavailability
of Drug Candidates. J. Med. Chem..

[48] Walters W. P., Murcko M. A. (2002). Prediction of ‘Drug-Likeness’. Adv. Drug Delivery Rev..

[49] Baell J. B., Holloway G. A. (2010). New Substructure Filters for Removal
of Pan Assay Interference
Compounds (PAINS) from Screening Libraries and for Their Exclusion
in Bioassays. J. Med. Chem..

[50] Brenk R., Schipani A., James D., Krasowski A., Gilbert I. H., Frearson J., Wyatt P. G. (2008). Lessons
Learnt from
Assembling Screening Libraries for Drug Discovery for Neglected Diseases. ChemMedchem.

[51] Hann M., Hudson B., Lewell X., Lifely R., Miller L., Ramsden N. (1999). Strategic Pooling of Compounds for High-Throughput
Screening. J. Chem. Inf. Comput. Sci..

[52] Hao G., Dong Q., Yang G. (2011). A comparative
study on the constitutive
properties of marketed pesticides. Mol. Inf..

[53] Adamczyk J., Poziemski J., Siedlecki P. (2025). ApisTox: a new benchmark dataset
for the classification of small molecules toxicity on honey bees. Sci. Data.

[54] Allen T. E., McDonagh J. L., Broncel M., Bryant C. J., Incarnato D., Vasudevan A., Khan R. T. (2024). Physicochemical principles driving
small molecule binding to RNA. bioRxiv.

[55] Galli S. (2021). Feature-engine:
A Python Package for Feature Engineering for Machine Learning. J. Open Source Software.

[56] Raymond J. W., Gardiner E. J., Willett P. (2002). RASCAL: Calculation of Graph Similarity
using Maximum Common Edge Subgraphs. Comput.
J..

[57] Hussain, J. Fraggle - A New Similarity Searching Algorithm. RDKit User Group Meeting; implementation in the RDKit, 2013. https://github.com/rdkit/rdkit.

[58] Ralaivola L., Swamidass S. J., Saigo H., Baldi P. (2005). Graph Kernels for Chemical
Informatics. Neural Networks.

[59] Bemis G. W., Murcko M. A. (1996). The Properties of
Known Drugs. 1. Molecular Frameworks. J. Med.
Chem..

[60] Ashton M., Barnard J., Casset F., Charlton M., Downs G., Gorse D., Holliday J., Lahana R., Willett P. (2002). Identification
of Diverse Database Subsets Using Property-Based and Fragment-Based
Molecular Descriptions. Quant. Struct.-Act.
Relat..

[61] Kim S., Chen J., Cheng T., Gindulyte A., He J., He S., Li Q., Shoemaker B. A., Thiessen P. A., Yu B., Zaslavsky L., Zhang J., Bolton E. E. (2023). PubChem 2023 Update. Nucleic Acids Res..

[62] Ke, G. ; Meng, Q. ; Finley, T. ; Wang, T. ; Chen, W. ; Ma, W. ; Ye, Q. ; Liu, T.-Y. LightGBM: A Highly Efficient Gradient Boosting Decision Tree. Advances in neural information processing systems. NeurIPS 2017.

[63] Sheridan R. P., Singh S. B., Fluder E. M., Kearsley S. K. (2001). Protocols
for Bridging
the Gap between Virtual Screening and High Throughput Screening. J. Chem. Inf. Comput. Sci..

[64] Truchon J.-F., Bayly C. I. (2007). Evaluating Virtual
Screening Methods: Good and Bad
Metrics for the “Early Recognition” Problem. J. Chem. Inf. Model..

[65] Wang S., Witek J., Landrum G. A., Riniker S. (2020). Improving Conformer
Generation for Small Rings and Macrocycles Based on Distance Geometry
and Experimental Torsional-Angle Preferences. J. Chem. Inf. Model..

[66] Gramatica P. (2007). Principles
of QSAR Models Validation: Internal and External. QSAR Comb. Sci..

[67] Wolf, T. ; Debut, L. ; Sanh, V. ; Chaumond, J. ; Delangue, C. ; Moi, A. ; Cistac, P. ; Rault, T. ; Louf, R. ; Funtowicz, M. HuggingFace’s Transformers: State-of-the-Art Natural Language Processing. arXiv. 2019

[68] Huang, K. ; Fu, T. ; Gao, W. ; Zhao, Y. ; Roohani, Y. ; Leskovec, J. ; Coley, C. W. ; Xiao, C. ; Sun, J. ; Zitnik, M. Therapeutics Data Commons: Machine Learning Datasets and Tasks for Drug Discovery and Development. arXiv. 2021.

[69] Dwivedi, V. P. ; Rampášek, L. ; Galkin, M. ; Parviz, A. ; Wolf, G. ; Luu, A. T. ; Beaini, D. Long Range Graph Benchmark. Proceedings of the 36th International Conference on Neural Information Processing Systems; ACM, 2022.

[70] van
Tilborg D., Alenicheva A., Grisoni F. (2022). Exposing the Limitations
of Molecular Machine Learning with Activity Cliffs. J. Chem. Inf. Model..

[71] McKinney, W. Data Structures for Statistical Computing in Python Proceedings Of The 9th Python In Science Conference SciPy 2010 56–61

[72] Bjerrum, E. J. ; Bachorz, R. A. ; Bitton, A. ; Choung, O. H. ; Chen, Y. ; Esposito, C. ; Ha, S. V. ; Poehlmann, A. Scikit-Mol brings cheminformatics to Scikit-Learn. ChemRxiv 2023.

[73] Karim A., Lee M., Balle T., Sattar A. (2021). CardioTox net: a robust predictor
for hERG channel blockade based on deep learning meta-feature ensembles. J. Cheminf..

[74] National Center for Biotechnology Information PubChem Substructure Fingerprint (V1.3), 2009. https://ftp.ncbi.nlm.nih.gov/pubchem/specifications/pubchem_fingerprints.pdf.

[75] Bajusz D., Rácz A., Héberger K. (2015). Why Is Tanimoto Index an Appropriate
Choice for Fingerprint-Based Similarity Calculations?. J. Cheminf..

[76] Chandrasekhar V., Rajan K., Kanakam S. R. S., Sharma N., Weißenborn V., Schaub J., Steinbeck C. (2025). COCONUT 2.0:
a comprehensive overhaul
and curation of the collection of open natural products database. Nucleic Acids Res..

[77] Zdrazil B., Felix E., Hunter F., Manners E. J., Blackshaw J., Corbett S., De Veij M., Ioannidis H., Lopez D. M., Mosquera J. F. (2024). The ChEMBL Database
in 2023: a drug discovery platform spanning multiple bioactivity data
types and time periods. Nucleic Acids Res..

[78] Mcule.com Mcule: Online Drug Discovery Platform and Purchasable Compound Database. https://mcule.com, Accessed 2026.

[79] Seidl, P. ; Vall, A. ; Hochreiter, S. ; Klambauer, G. Enhancing Activity Prediction Models in Drug Discovery with the Ability to Understand Human Language. In Proceedings Of The 40th International Conference On Machine Learning (ICML); ICML, 2023, 202.

[80] Burns, J. W. Deep Learning Foundation Models from Classical Molecular Descriptors. arXiv. 2025 10.1021/acs.jcim.6c01546 42734497

[81] Praski, M. ; Adamczyk, J. ; Czech, W. Benchmarking pretrained molecular embedding models for molecular representation learning. arXiv. 2025.

[82] Joeres R., Blumenthal D. B., Kalinina O. V. (2025). Data splitting to avoid information
leakage with DataSAIL. Nat. Commun..

